# Supplementary material for: Development of Highly Efficient Universal Pneumocystis Primers and Their Application in Investigating the Prevalence and Genetic Diversity of Pneumocystis in Wild Hares and Rabbits
Source: J Fungi (Basel). 2024 May 15;10(5):355. doi: 10.3390/jof10050355 (PMC11121927; doi:10.3390/jof10050355)
Supplement: Supplementary file 1 [file jof-10-00355-s001.zip › Figure S1_mtLSU alignment and primers.pdf]

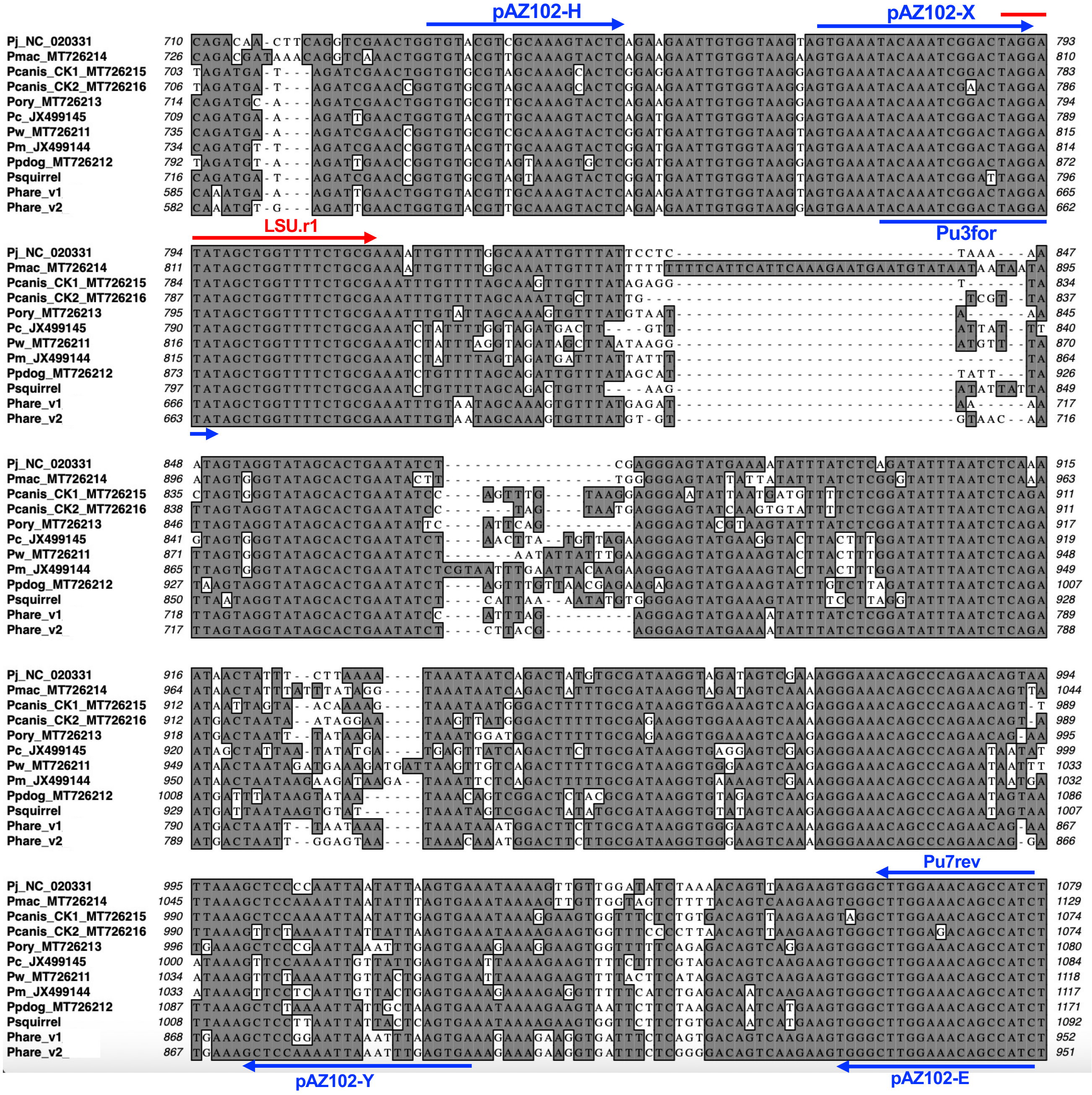

This portion (corresponding to nucleotides 1080 to 1191 in Pj) is omitted to reduce the space

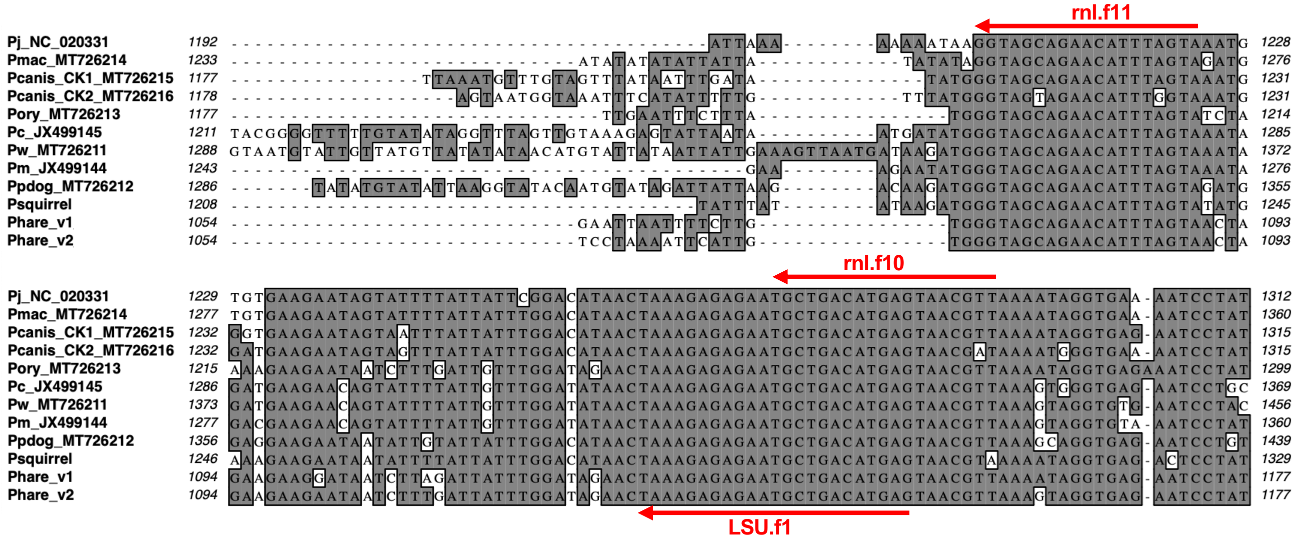

Supplemental Figure S1. Alignment of mitochondrial large-subunit (mtLSU) rRNA sequences of different *Pneumocystis* species and strains. The locations of the previously used universal *Pneumocystis* primer pairs pAZ102-E – pAZ102-H [34], pAZ102-X – pAZ102-Y [38] and Pu3for – Pu7rev [33] are indicated by arrows along with their names in blue. The sequences of these primers are identical to the respective regions in *P. jirovecii* (Pj). Selected new primers designed in this study are indicated in red. Primer Pu3Y.f is identical to Pu3for except for the replacement of A at the 10th position (from the 5′ end) by a degenerate nucleotide R to compensate the variation between A and G (Table S2). Highly conserved sequences are highlighted in dark shadow. Dashes represents gaps introduced to optimize alignment. GenBank accession numbers are indicated after the names of *Pneumocystis* species/strains abbreviated as follows: Pj, *P. jirovecii;* Pmac, *P. macacae;* Pcanis_CK1, *P. canis* strain CK1; Pcanis_CK2, *P. canis* strain CK2; Pory, *P. oryctolagi;* Pc, *P. carinii;* Pw, *P. wakefieldiae;* Pm, *P. murina,* Ppdog, *Pneumocystis ludoviciani* from prairie dogs [Table S6]; Psquirrel, *Pneumocystis* from tree squirrels; Phare_v1, *P.* sp. *'townsendii'* variant 1 from hares; Phare_v2, *P.* sp. *'townsendii'* variant 2 from hares. GenBank accession numbers for the last 3 sequences are PP484879, PP477327 and PP477328. Numbers on the two ends of each sequence correspond to the nucleotide positions of the full-length mtLSU gene in GenBank.
