# Supplementary material for: Development of Highly Efficient Universal Pneumocystis Primers and Their Application in Investigating the Prevalence and Genetic Diversity of Pneumocystis in Wild Hares and Rabbits
Source: J Fungi (Basel). 2024 May 15;10(5):355. doi: 10.3390/jof10050355 (PMC11121927; doi:10.3390/jof10050355)
Supplement: Supplementary file 1 [file jof-10-00355-s001.zip › Figure S2_mtSSU alignment and primers.pdf]

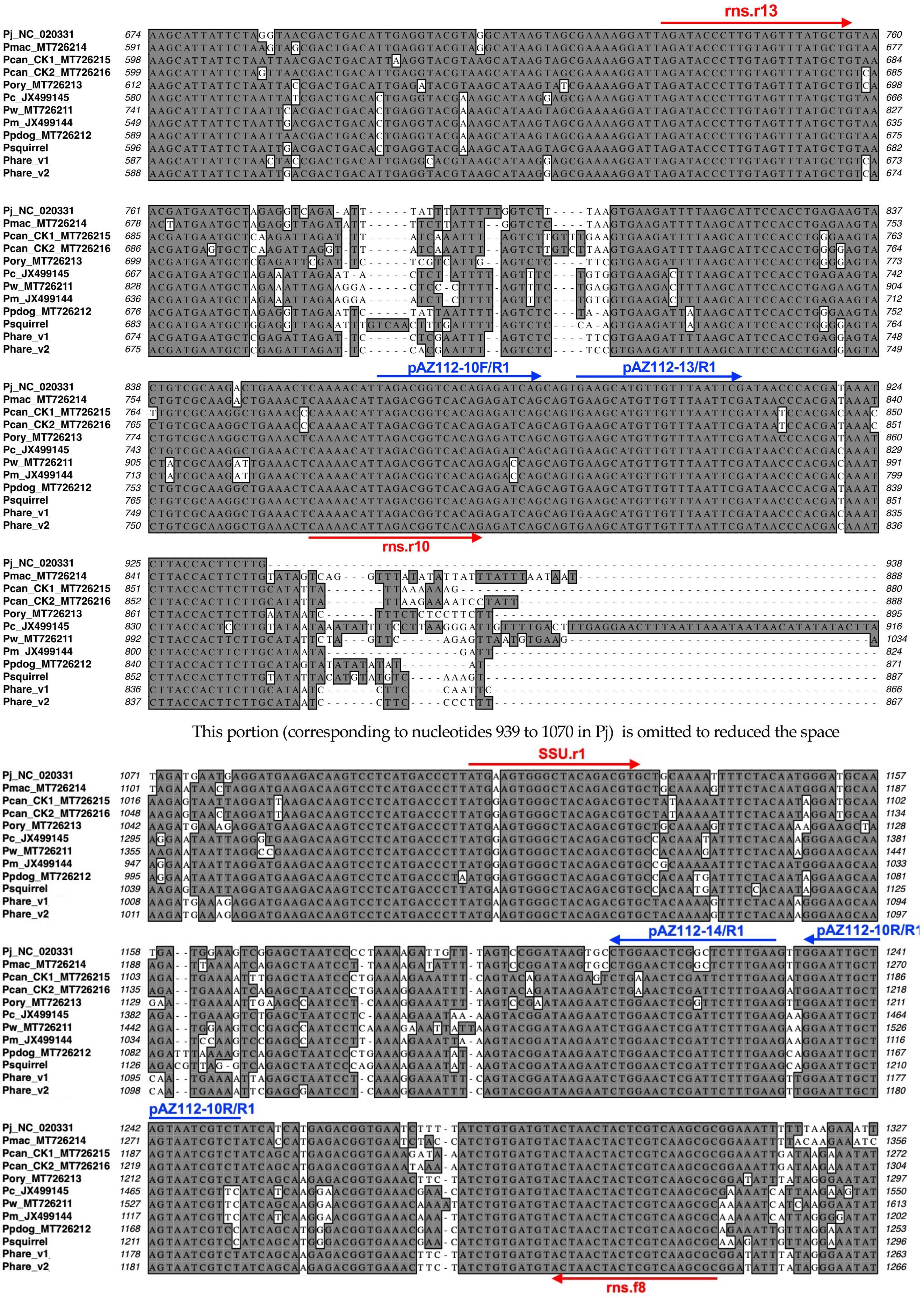
rns.r13
Pj_NC_020331
Pmac_MT726214
Pcan_CK1_MT726215
Pcan_CK2_MT726216
Pory_MT726213
Pc_JX499145
Pw_MT726211
Pm_JX499144
Ppdog_MT726212
Psquirrel
Phare_v1
Phare_v2
pAZ112-10F/R1
pAZ112-13/R1
rns.r10
This portion (corresponding to nucleotides 939 to 1070 in Pj) is omitted to reduced the space
SSU.r1
pAZ112-14/R1
pAZ112-10R/R1
rns.f8

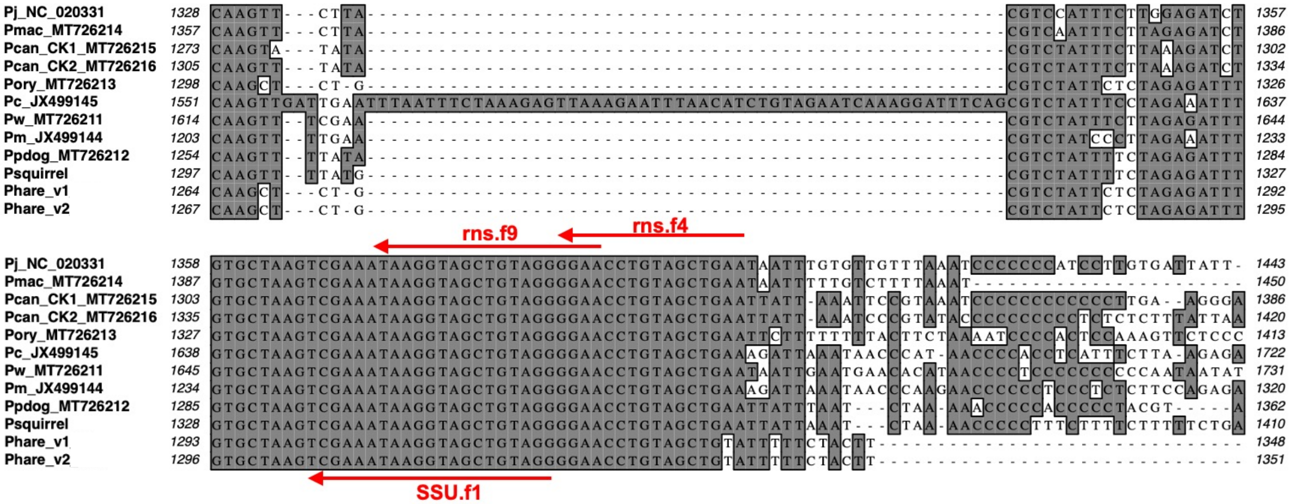

Supplemental Figure S2. Alignment of mitochondrial small-subunit (mtSSU) rRNA sequences of different *Pneumocystis* species and strains. Highly conserved sequences are highlighted in dark shadow. Dashes represents gaps introduced to optimize alignment. New primers designed in this study are indicated in red. The locations of the previously used universal *Pneumocystis* primers [38] were indicated in blue. The sequences of primers pAZ112-10F/R1 and pAZ112-13/R1 are identical to the respective regions in *P. jirovecii* (Pj), primer pAZ112-10R/R1 sequence is identical to the respective regions in *P. carinii* (Pc), and primer pAZ112-14/R1 contains a degenerate nucleotide (Y) at two positions (5'-TTCAAAGAAYCGAGTTYCAG-3'), which were omitted in the reference [37]. GenBank accession numbers are indicated after the names of *Pneumocystis* species/strains abbreviated as follows: Pj, *P. jirovecii;* Pmac, *P. macacae;* Pcan_CK1, *P. canis* strain CK1; Pcan_CK2, *P. canis* strain CK2; Pory, *P. oryctolagi;* Pc, *P. carinii;* Pw, *P. wakefieldiae;* Pm, *P. murina,* Ppdog, *Pneumocystis ludoviciani* from prairie dogs (Table S6); Psquirrel, *Pneumocystis* from squirrels; Phare_v1, *P.* sp. *'townsendii'* variant 1 from hares; Phare_v2, *P.* sp. *'townsendii'* variant 2 from hares. GenBank accession numbers for the last 3 sequences are PP484879, PP477339 and PP477340. Numbers on the two ends of each sequence correspond to the nucleotide positions of the full-length mtLSU gene in GenBank.
