# Supplementary material for: Development of Highly Efficient Universal Pneumocystis Primers and Their Application in Investigating the Prevalence and Genetic Diversity of Pneumocystis in Wild Hares and Rabbits
Source: J Fungi (Basel). 2024 May 15;10(5):355. doi: 10.3390/jof10050355 (PMC11121927; doi:10.3390/jof10050355)
Supplement: Supplementary file 1 [file jof-10-00355-s001.zip › Figure S3_ITS alignment and primers.pdf]

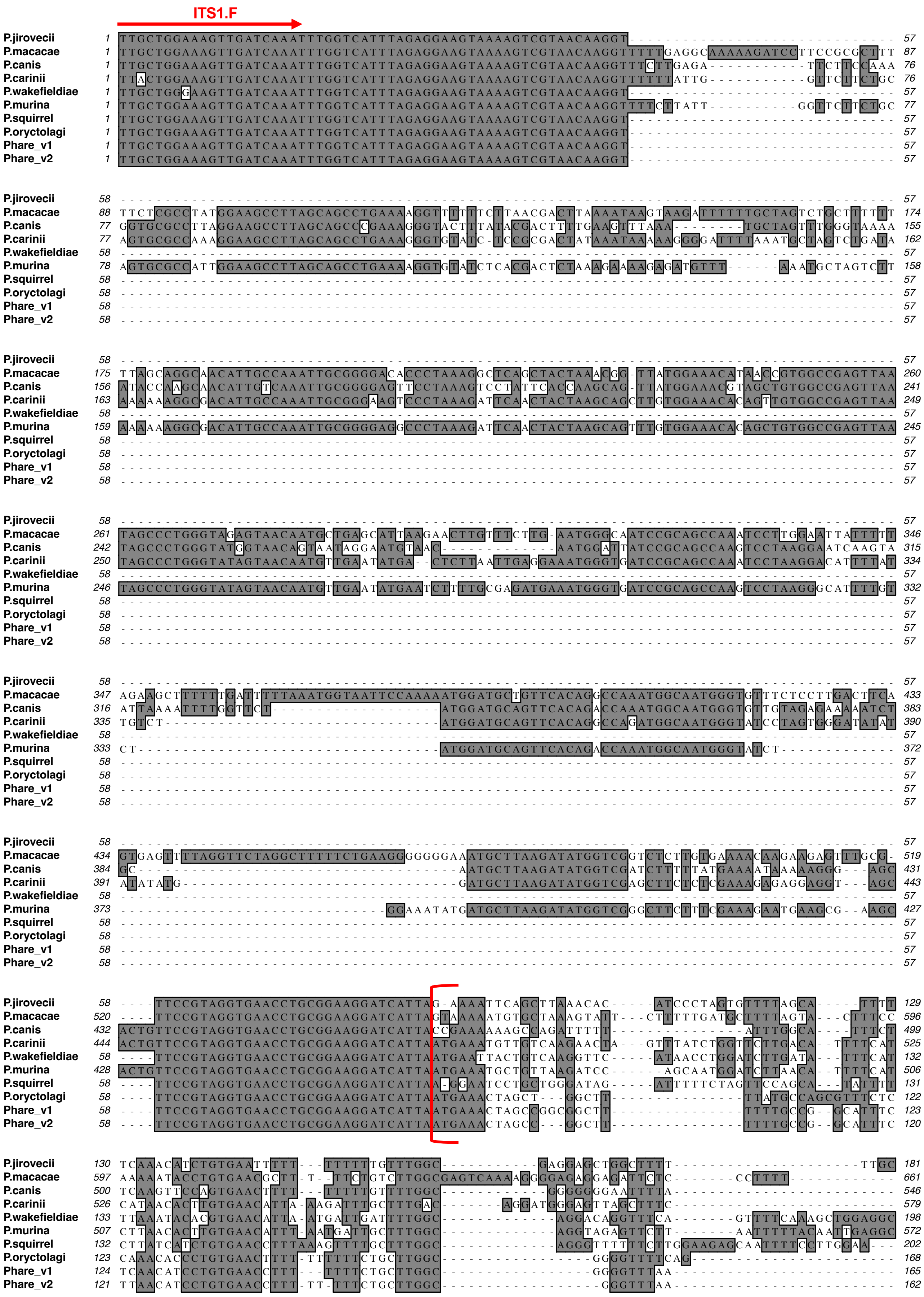
ITS1.F
P.jirovecii
P.macacae
P.canis
P.carinii
P.wakefieldiae
P.murina
P.squirrel
P.oryctolagi
Phare_v1
Phare_v2

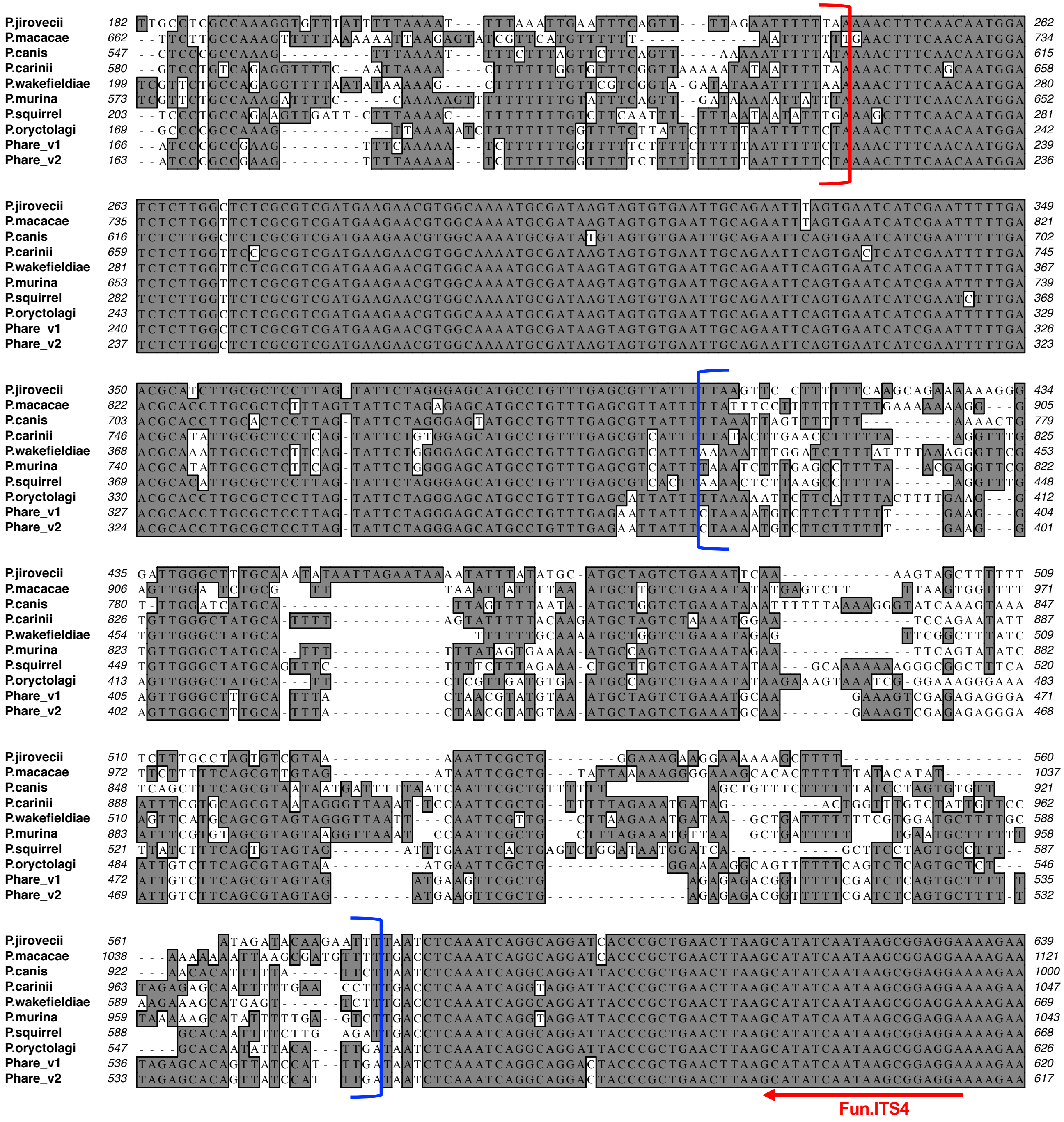

Supplemental Figure S3. Alignment of nuclear internal transcribed spacer 1-5.8S rRNA-internal transcribed spacer 2 (ITS1-5.8S-ITS2) sequences of different *Pneumocystis* species and strains. Highly conserved sequences are highlighted in dark shadow. Dashes represents gaps introduced to optimize alignment. The ITS1 region is indicated by red square brackets while the ITS2 region is indicated by blue square brackets. The locations of the primer pair ITS1.F-Fun.ITS4, which showed high amplification efficiency (Figure 1D), are indicated by arrows. The sequences of these primers are listed in Table S2. GenBank accession numbers for all sequences are listed in Table S6. P.squirrel, *Pneumocystis* from tree squirrels; Phare_v1, *P.* sp. *'townsendii'* variant 1 from hares; Phare_v2, *P.* sp. *'townsendii'* variant 2 from hares.
