# Supplementary material for: Development of Highly Efficient Universal Pneumocystis Primers and Their Application in Investigating the Prevalence and Genetic Diversity of Pneumocystis in Wild Hares and Rabbits
Source: J Fungi (Basel). 2024 May 15;10(5):355. doi: 10.3390/jof10050355 (PMC11121927; doi:10.3390/jof10050355)
Supplement: Supplementary file 1 [file jof-10-00355-s001.zip › Figure S4_Phare mtLSU variants.pdf]

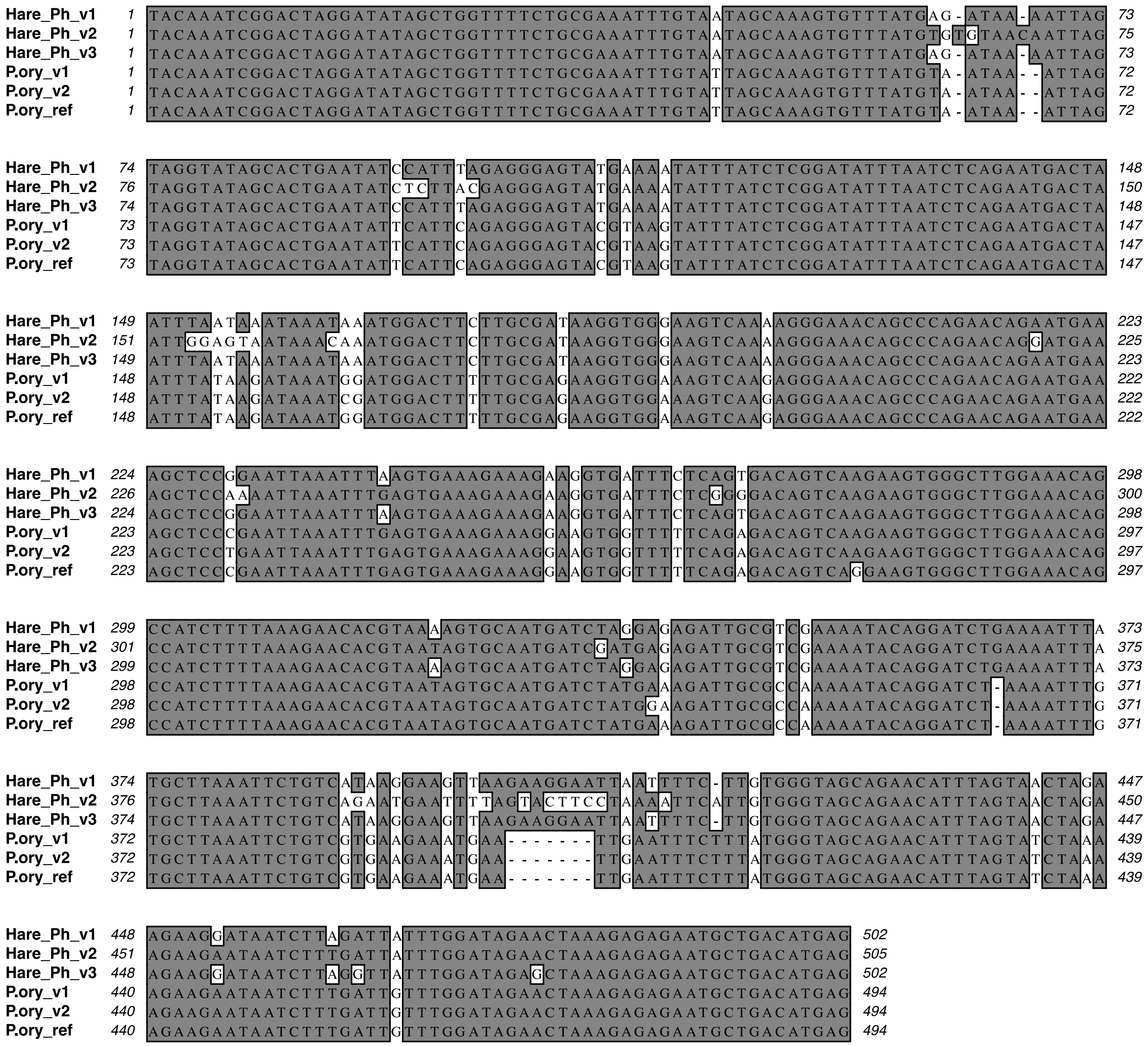

Supplemental Figure S4. Variation of mitochondrial large-subunit rRNA (mtLSU) sequences of *Pneumocystis* variants in hares and rabbits aligned with *P. oryctolagi* reference sequence (P.ory_ref, from Michigan, USA. GenBank accession no. MT726213). Identical nucleotides among multiple variants are highlighted in dark shadow. The 3 variants of *P.* sp. *'townsendii'* from hares are represented by Hare_Ph_v1 to Hare_Ph_v3. The 2 variants *of P. oryctolagi* are represented by P.ory_v1 and P.ory_v2. All sequences determined in this study have been deposited into GenBank with accession nos. available from Table S6.
