# Supplementary material for: Development of Highly Efficient Universal Pneumocystis Primers and Their Application in Investigating the Prevalence and Genetic Diversity of Pneumocystis in Wild Hares and Rabbits
Source: J Fungi (Basel). 2024 May 15;10(5):355. doi: 10.3390/jof10050355 (PMC11121927; doi:10.3390/jof10050355)
Supplement: Supplementary file 1 [file jof-10-00355-s001.zip › Figure S5_Pory and Phare dhps.pdf]

Sequence	Start	Nucleotides	End
Phare_v1	1	TATGAATCAGAGCCTATGTATTTTGAAAATCAGCCAATATTTTATAATGCTGTATGTAAGGTATATAGTATTTTTCGTTTTAATT	85
Phare_v2	1	TATGAATCAGAGCCTATGTATTTTGAAAATCAGCCAATATTTTATAATGCTGTATGTAAGGTATATAGTATTTTTCATTTTAATT	85
P.oryctolagi	1	TATGAATCGGAGCCTATGTATTTTGAAAATCAACCACTATTTTATAATGCTGTATGTAAGGTATATAGTATTTTTCATTTTAATT	85
Phare_v1	86	GATAATTTTGTTTATTGTTTAAGGTTGAGACTTCTTTAAATCCTCAACAGCTTCTTATTGAATTACAAACAATTGAAAAGGAGCT	170
Phare_v2	86	GACAATTTTGTTTATTGTTTAAGGTAGAGACTTCTTTAAATCCTCAACAGCTTCTTATTGAATTACAAACAATTGAAAAGGAGCT	170
P.oryctolagi	86	GATAATTTTATTTATTGTTTAAGGTTGAGACTTCTTTAAATCCTCAACAGCTTCTTATTGAGTTGCAAACAATTGAGAAGGAGCT	170
Phare_v1	171	TGGACGTATTAAGACTATTGATAAAGGTCCTCGATGCATTGATTTAGATATTATTTTTTTTGGTAGACAAATAATTAATTCTGAA	255
Phare_v2	171	TGGACGTATTAAGACTATTGATAAAGGTCCTCGATGCATTGATTTAGATATTATTTTTTTTGGTAGACAAATAATTAATTCTGAA	255
P.oryctolagi	171	TGGACGTATTAAGACTATTGATAAAGGTCCTCGATGCATTGATTTAGATATTATTTTTTTTGGTAGACAAATAATTAACTCTGAA	255
Phare_v1	256	TCATTAATTGTTCCTCATCTCAGAATACTTGAACGTCCATTTGTTCTAAAACCGCTTTCTGAGTAAATGATAATATAGATAAGGT	340
Phare_v2	256	TCATTAATTGTTCCTCATCCTAGAATACTTGAACGTCCATTTGTTCTAAAACCGCTTTCTGAGTAAATGATAATATAGATAAGGT	340
P.oryctolagi	256	TCACTATTTGTCCCTCATCCTAGGATACTTGAACGTCCATTTGTTCTAAAACCACTTTCTGAGTAAATAGTAGTATAGATAAGAT	340
Phare_v1	341	AAATATTTTTCTAATTATAAATTAGTATATCGAGTGATATTATTCATCCTGTTACCGGGTTATCTATAGTTTCATATCTTAGAAA	425
Phare_v2	341	AAATGTTTTTCTAATTATAAATTAGTATATCGAGTGATATTATTCATCCTGTTACCGGGTTATCTATAGTTTCATATCTTAGAAA	425
P.oryctolagi	341	AATTGTTTTTCTAATTATAAATTAGTATATCGAATGATATTATTCATCCTGTTACTGGATTATCTATAGTTTCGTATCTTAAAAA	425
Phare_v1	426	AATAGATAATCTTAATATTAAACCAGTTTTGCCATTTTTATATAAAAATAAAAGTATAAATTTTAGTTCTGGGTCCTATAAAGCA	510
Phare_v2	426	AATAGATAATCTTAATATTAGACCAGTTTTACCATTTTTATATAAAAATAAAAGTATAAATTTTAGTTTTGGGTCCTATAAAGTA	510
P.oryctolagi	426	AACAGATAGTCTTAATATTAAACCAGTTTTGCCATTTTTATATAAGAATAAAAGTATAAATTTCAGTTTTGGATCTTATAAATCA	510
Phare_v1	511	CCCACACATATTATGGCTATTTTGAATCTCACTCCTGATTCTTTTTCTGATGGTGGTATTCATTCATATGGTTCTATATTAATGG	595
Phare_v2	511	CCTACACATATTATGGCTATTTTGAATCTCACTCCTGATTCTTTTTCTGATGGTGGTATTCATTCATATGGTTCTATATTGATGG	595
P.oryctolagi	511	CCTACACATATTATGGCTATTTTGAATCTCACTCCTGACTCTTTTTTTGATGGTGGCATTCATTCATATGATTCTATATTGATAG	595
Phare_v1	596	ATGCTGAGAAGTTTATAAAAGCTGGTGCAACAATAATTGATATTGGTGGACAGTCTACAAGGCCTGGTTCTCATATTGTTTCTAC	680
Phare_v2	596	ATGCTGAGAAGTTTATAAAAGCTGGTGCAACAATAATTGATATTGGTGGACAGTCTACAAGGCCTGGTTCTCATATTGTTTCTAC	680
P.oryctolagi	596	ATGCAGAGAAGTTTGTAAAAGCTGGTGCAACAATAATTGATATTGGTGGACAGTCTACAAGGCCTGGTTCTCATATTATTTCTAT	680
Phare_v1	681	AGATGAAGAAATATCTCGAGTTATTCCTGCTATAAAACGACTTTTAGAAACATATCCTGATATTTTAGTAAGTATAGATACATTT	765
Phare_v2	681	AGATGAAGAAATATCTCGAGTTATTCCTGCTATAAAACGACTTTTAGAAACATATCCTGATATTTTAGTAAGTATAGATACATTT	765
P.oryctolagi	681	AGAGGAAGAAATTTCTCGGGTTGTTCCTGCTATAAAACGACTTTTAAAAACATATCCCGATATTTTAGTAAGTATAGATACATTT	765
Phare_v1	766	CGTTCTGAAGTTGCAGAAGAAGCAATTAGGGCCGGTGCTAGTCTTGTTAATGATGTAACAGGAGGGAAATATGACCCGAAAATGT	850
Phare_v2	766	CGTTCTGAAGTTGCAGAAGAAGCAATTAGGGCCGGTGCCTGTCTTGTTAATGATGTAACAGGAGGGAAATATGACCCAAAAATGT	850
P.oryctolagi	766	CGTTCTGAAGTTGCAGAAGAAGCAATTAGAGCCGGTGCTTGTCTTGTTAATGATGTAACGGGAGGGAAATATGATCCGAAAATGT	850
Phare_v1	851	TTAAAACTGTGGCTAAATTTCAAGTTCCAATATGTATAATGCATATGAGAGGCGATTTTTTAACCATGGATAATTTAACAGATTA	935
Phare_v2	851	TTAAGACTGTGGCTAAATTTCAAGTTCCAATATGTATAATGCATATGAGAGGCGATTTTTTAACCATGGATAACTTAACAGATTA	935
P.oryctolagi	851	TTAAAACTGTCGCTAAATTTCAAGTTCCGATATGTATAATGCATATGAGAGGCGATTTTTTAACCATGGATAATTTAACAGATTA	935
Phare_v1	936	TGGTGCTGATATTGTAAAGCAGATCACTATAGAGTTAGAAGAGTTGCTTATTTGTGCCGAAAATTCGGGTATTCCTCGCTGGAAT	1020
Phare_v2	936	TGGTACTGATATTGTAAAGCAGATCACTATAGAGTTAGAAGAATTGCTTATTTGTGCCGAAAATTCGGGTATTCCTCGCTGGAAT	1020
P.oryctolagi	936	TGGTACTGATATTGTAAAGCAAATCACTGTGGAGTTAGAAGAATTGCTTATTTGTGCCGAAAATTCGGGTATTCCTCGTTGGAAT	1020
Phare_v1	1021	ATTATTTTAGATCCCGGGTTAGGATTTGCTAAAACTTCATATCAGAATATAGAGTTATTGAGAAGGTTTAATGAATTAAAATCTC	1105
Phare_v2	1021	ATTATTTTAGATCCCGGGTTAGGATTTGCTAAAACTTCATATCAGAATATAGAGTTATTGAGAAGGTTTAATGAATTAAAATCTC	1105
P.oryctolagi	1021	ATTATTTTAGATCCTGGGTTAGGATTTGCTAAAACTTCATATCAGAATATAGAGTTATTAAGAAGGTTTAATGAATTAAAATCTC	1105
Phare_v1	1106	AGCCTTGTTTTAGAGGTTTGCCCTGGTTGCTTGGTCCTAGTCGAAAAAGATTTACAGGCTGTATTACAGGGGATATTGTTCCAAG	1190
Phare_v2	1106	AGCCTTGTTTTAGAGGTCTTCCCTGGTTGCTTGGTCCTAGTCGAAAAAGATTTACAGGCTGTATTACAGGGGATATTGTTCCAAG	1190
P.oryctolagi	1106	AGAGTTGCTTCAGTGGTTTACCCTGGTTGCTCGGTCCAAGTCGAAAAAGATTTACAGGATGTGTTACAGGGGATATTGTTCCAAG	1190
Phare_v1	1191	AGATAGGATTTGGGGCACGGCTGCTGCTGTTACTGCATCTATTTTAGGAGGATGTGATATTATACGGATTCATGATGTTTTTGAA	1275
Phare_v2	1191	AGATAGGATTTGGGGCACGGCTGCTGCTGTTACTGCATCTATTTTAGGAGGATGTGATATTATACGGATTCATGATGTTTTTGAA	1275
P.oryctolagi	1191	AGATAGAATTTGGGGTACGGCTGCTGCTGTTACTGCATCTATTTTAGGAGGATGTGATATTATACGGGTTCATGATGTTTTTGAA	1275
Phare_v1	1276	ATGTGCAAGGTGTTAAAGACTTCAGATGCTAT	1307
Phare_v2	1276	ATGTGCAAGGTGTTAAAGACTTCAGATGCTAT	1307
P.oryctolagi	1276	ATGTGTAAAGTTTCAAGGATTTCAGATGCTAT	1307

Supplemental Figure S5. Nucleotide sequences of the dihydropteroate synthase (*dhps*) gene (enclosed in square brackets) and its upstream dihydro-6-hydroxymethylpterin pyrophosphokinase (*pppk*) gene of *Pneumocystis* from hares and rabbits. Highly conserved sequences are highlighted in dark shadow. Nucleotide variations resulting tin amino acid changes in the *dhps* gene are indicated by red squares. Phare_v1, *P.* sp. *'townsendii'* variant 1; Phare_v2, *P.* sp. *'townsendii'* variant 2 . Numbers on the two ends of each sequence correspond to the nucleotide positions of the full-length sequence of the region amplified by PCR deposited in GenBank with accession no. listed in Table S6.
