# Supplementary material for: Development of Highly Efficient Universal Pneumocystis Primers and Their Application in Investigating the Prevalence and Genetic Diversity of Pneumocystis in Wild Hares and Rabbits
Source: J Fungi (Basel). 2024 May 15;10(5):355. doi: 10.3390/jof10050355 (PMC11121927; doi:10.3390/jof10050355)
Supplement: Supplementary file 1 [file jof-10-00355-s001.zip › Figure S6_dhps and rDNA trees.pdf]

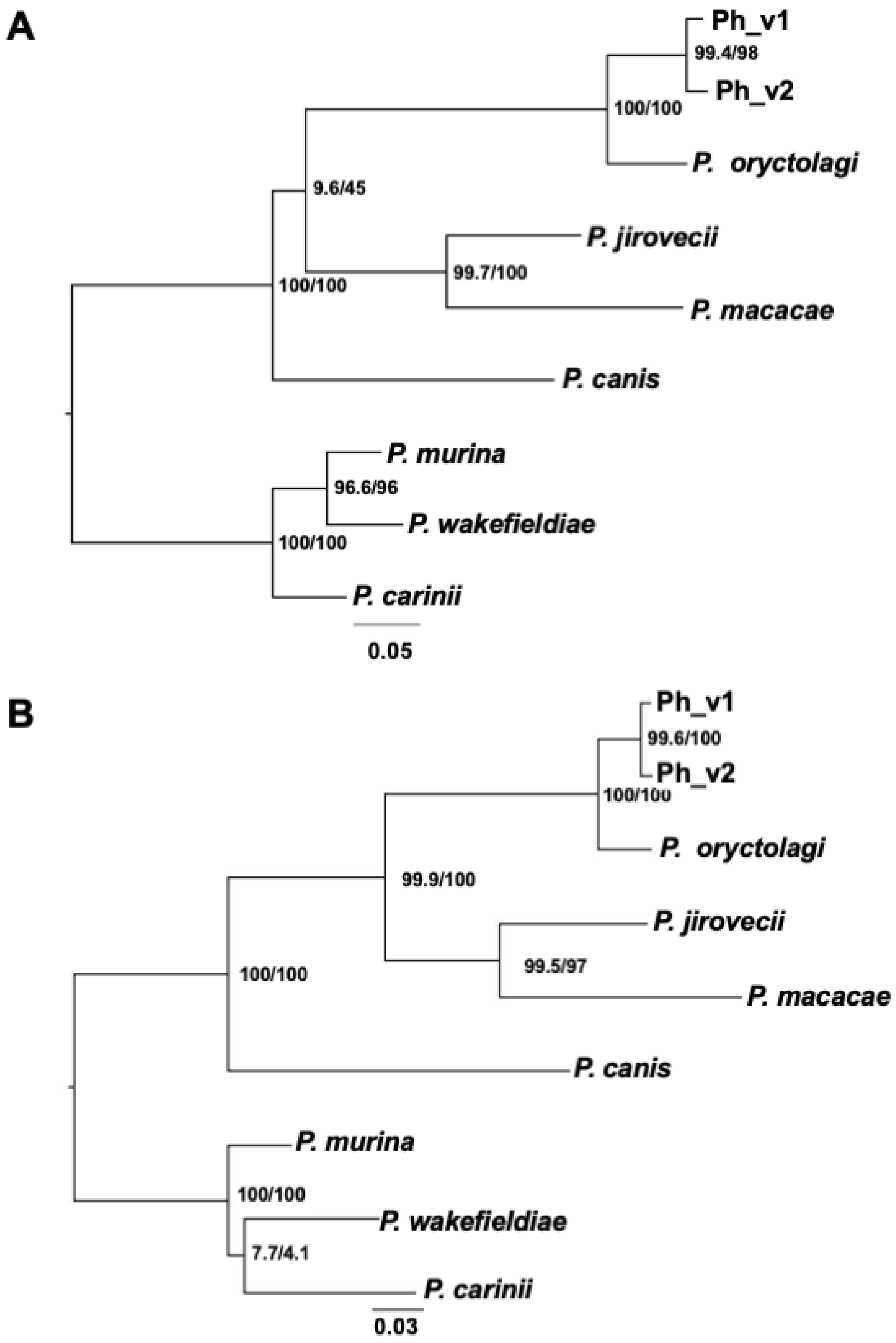

Supplemental Figure S6. Maximum likelihood phylogenetic trees of *Pneumocystis* from hares and other mammals based on the dihydropteroate synthase (*dhps*) gene (**A**) and nuclear rRNA operon (**B**). The trees were constructed using nucleotide sequences from each *Pneumocystis* species or strain, with GenBank accession numbers listed in Table S6. The order of species and strains in panel B was rotated, without changing the clustering relationships, to facilitate visual comparison with other trees. Numbers at each node are SH-aLRT support (%)/ultrafast bootstrap support (%). The 2 variants of *P.* sp. *'townsendii'* from hares identified in this study are represented by Ph_v1 and Ph_v2.
