# Supplementary material for: Development of Highly Efficient Universal Pneumocystis Primers and Their Application in Investigating the Prevalence and Genetic Diversity of Pneumocystis in Wild Hares and Rabbits
Source: J Fungi (Basel). 2024 May 15;10(5):355. doi: 10.3390/jof10050355 (PMC11121927; doi:10.3390/jof10050355)
Supplement: Supplementary file 1 [file jof-10-00355-s001.zip › Table S1_Metadata for hares and rabbits.pdf]

Supplemental Table S1. Metadata for hares and rabbits used in this study.

Animal number	Date Carcass Collected	Date Sample Collected	Animal Species	Sex (Male=1, Female=2)	mtSSU PCR Results	mtLSU PCR Results	Combined PCR Results	Genotypes	dhps PCR Results	ITS1-5.8S-ITS2 PCR Results
HR20-001	?-Dec-2019	20-Jan-2020	*Lepus townsendii*	2	+	+	+	v1	–	–
HR20-002	30-Dec-2019	20-Jan-2020	*Lepus townsendii*	1	+	+	+	v1	–	+
HR20-003	?-Dec-2019	20-Jan-2020	*Lepus townsendii*	2	+	+	+	v1	–	+
HR20-004	28-Dec-2019	22-Jan-2020	*Lepus townsendii*	1	+	+	+	v1	+	+
HR20-005	?-Dec-2019	22-Jan-2020	*Lepus townsendii*	2	+	+	+	v1	–	–
HR20-006	15-Dec-2019	22-Jan-2020	*Lepus townsendii*	1	+	+	+	v1	+	+
HR20-007	29-Dec-2019	23-Jan-2020	*Lepus townsendii*	1	+	+	+	v1	–	–
HR20-008	10-Dec-2019	23-Jan-2020	*Lepus townsendii*	1	+	+	+	v1	–	–
HR20-009	20-Dec-2019	23-Jan-2020	*Lepus townsendii*	1	+	+	+	v1	+	+
HR20-010	Unknowm	24-Jan-2020	*Lepus townsendii*	1	+	+	+	v1	–	–
HR20-011	16-Dec-2019	24-Jan-2020	*Lepus townsendii*	1	+	+	+	v1	+	–
HR20-012	19-Dec-2019	24-Jan-2020	*Lepus townsendii*	2	–	–	–	n/a	–	–
HR20-013	30-Dec-2019	24-Jan-2020	*Lepus townsendii*	1	+	+	+	v3	–	+
HR20-014	?-Dec-2019	24-Jan-2020	*Lepus townsendii*	1	+	+	+	v1	+	+
HR20-015	Unknowm	7-Jan-2020	*Lepus townsendii*	1	+	+	+	v1	+	+
HR20-016	11-Dec-2019	27-Jan-2020	*Lepus townsendii*	2	–	–	–	n/a	–	–
HR20-017	24-Jan-2020	27-Jan-2020	*Lepus townsendii*	2	+	+	+	v1	–	+
HR20-018	?-?-2020	27-Jan-2020	*Lepus townsendii*	2	+	+	+	v1	+	+
HR20-019	21-Jan-2020	28-Jan-2020	*Lepus townsendii*	2	+	+	+	v1	–	+
HR20-020	22-Jan-2020	28-Jan-2020	*Lepus townsendii*	2	+	+	+	v1	+	+
HR20-021	19-Jan-2020	28-Jan-2020	*Lepus townsendii*	2	–	–	–	n/a	–	–
HR20-022	25-Jan-2020	31-Jan-2020	*Lepus townsendii*	1	+	+	+	v1	–	–
HR20-023	28-Jan-2020	31-Jan-2020	*Lepus townsendii*	1	+	+	+	v1	–	–
HR20-024	25-Jan-2020	3-Feb-2020	*Lepus townsendii*	2	+	+	+	v1	+	+
HR20-025	25-Jan-2020	3-Feb-2020	*Lepus townsendii*	1	+	+	+	v1	+	+
HR20-028	28-Jan-2020	4-Feb-2020	*Lepus townsendii*	2	+	+	+	v1	+	+
HR20-029	3-Feb-2020	12-Feb-2020	*Lepus townsendii*	2	+	+	+	v1	+	+
HR20-030	30-Jan-2020	12-Feb-2020	*Lepus townsendii*	1	+	+	+	v1	+	+
HR20-031	31-Jan-2020	12-Feb-2020	*Lepus townsendii*	2	+	+	+	v1	–	–
HR20-032	19-Feb-2020	26-Feb-2020	*Lepus townsendii*	1	+	+	+	v1	+	–
HR20-033	21-Feb-2020	26-Feb-2020	*Lepus townsendii*	1	+	+	+	v1	+	+
HR20-034	14-Feb-2020	26-Feb-2020	*Lepus townsendii*	1	+	+	+	v1	+	+
HR20-036	19-Feb-2020	27-Feb-2020	*Lepus townsendii*	2	+	+	+	v1	–	+
HR20-037	29-Feb-2020	4-Mar-2020	*Lepus townsendii*	1	+	+	+	v1	–	–
HR20-038	4-Mar-2020	4-Mar-2020	*Lepus townsendii*	1	–	–	–	n/a	–	+
HR20-039	25-Feb-2020	4-Mar-2020	*Lepus townsendii*	2	+	+	+	v1	+	+
HR20-041	5-Mar-2020	12-Mar-2020	*Lepus townsendii*	1	+	+	+	v1	–	+
HR20-043	3-Mar-2020	12-Mar-2020	*Lepus townsendii*	Unknowm	+	+	+	v1	+	+
HR20-044	1-Mar-2020	13-Mar-2020	*Lepus townsendii*	1	–	–	–	n/a	–	+
HR20-045	3-Mar-2020	13-Mar-2020	*Lepus townsendii*	2	+	+	+	v1	–	+
HR20-046	11-Mar-2020	17-Mar-2020	*Lepus townsendii*	2	+	+	+	v1	–	+
HR20-047	11-Mar-2020	17-Mar-2020	*Lepus townsendii*	1	+	+	+	v1	+	+
HR20-048	6-Mar-2020	13-Mar-2020	*Lepus townsendii*	Unknowm	+	–	+	v1	–	–
HR20-049	?-Mar-2020	17-Mar-2020	*Lepus townsendii*	2	+	+	+	v1	–	+
HR20-050	9-Mar-2020	19-Mar-2020	*Lepus townsendii*	2	–	–	–	n/a	–	–
HR20-051	9-Mar-2020	19-Mar-2020	*Lepus townsendii*	2	+	+	+	v1	–	–
HR20-052	9-Apr-2020	23-Jun-2020	*Lepus townsendii*	2	+	+	+	v1	–	–
HR20-053	7-Apr-2020	23-Jun-2020	*Lepus townsendii*	2	+	+	+	v1	–	–
HR20-055	3-Apr-2020	25-Jun-2020	*Lepus townsendii*	1	+	–	+	v1	–	–
HR20-056	30-Mar-2020	26-Jun-2020	*Lepus townsendii*	2	+	+	+	v1	–	–
HR20-057	27-Mar-2020	26-Jun-2020	*Lepus townsendii*	2	+	+	+	v1	–	–
HR20-058	Unknowm	13-Jul-2020	*Lepus townsendii*	2	+	+	+	v1	–	–
HR20-059	30-Mar-2020	13-Jul-2020	*Lepus townsendii*	1	–	–	–	n/a	–	–
HR20-060	18-Mar-2020	13-Jul-2020	*Lepus townsendii*	2	+	+	+	v1	+	+
HR20-061	21-Mar-2020	14-Jul-2020	*Lepus townsendii*	1	+	+	+	v1	+	–
HR20-062	22-Mar-2020	14-Jul-2020	*Lepus townsendii*	1	+	+	+	v1	+	–
HR20-064	Unknowm	15-Jul-2020	*Lepus townsendii*	1	+	+	+	v1	–	–
HR20-065	9-Apr-2020	15-Jul-2020	*Lepus townsendii*	1	+	+	+	v1	+	+
HR20-066	Unknowm	17-Jul-2020	*Lepus townsendii*	1	+	–	+	v1	–	+
HR20-067	25-Mar-2020	17-Jul-2020	*Lepus townsendii*	2	–	–	–	n/a	–	–
HR20-068	12-May-2020	27-Jul-2020	*Lepus townsendii*	2	+	+	+	v1	–	+
HR20-069	18-Mar-2020	27-Jul-2020	*Lepus townsendii*	1	+	+	+	v1	–	+
HR20-070	29-Mar-2020	28-Jul-2020	*Lepus townsendii*	1	–	–	–	v1	–	+
HR20-071	?-?-2020	28-Jul-2020	*Lepus townsendii*	2	+	+	+	v1	–	+
HR20-072	6-Jun-2020	30-Jul-2020	*Lepus townsendii*	2	+	+	+	v2	+	–
HR20-073	10-Jun-2020	30-Jul-2020	*Lepus townsendii*	2	+	+	+	v1	–	+
HR20-074	25-May-2020	30-Jul-2020	*Lepus townsendii*	1	+	+	+	v1	+	+
HR20-075	8-Jun-2020	31-Jul-2020	*Lepus townsendii*	2	+	+	+	v1	–	+

HR20-076	2-May-2020	31-Jul-2020	*Lepus townsendii*	2	+	–	+	v4	–	+
HR20-077	5-May-2020	4-Aug-2020	*Lepus townsendii*	1	+	+	+	v2	+	+
HR20-078	23-Apr-2020	4-Aug-2020	*Lepus townsendii*	1	–	–	–	n/a	–	–
HR20-079	19-May-2020	7-Aug-2020	*Lepus townsendii*	2	–	–	–	n/a	–	+
HR20-080	7-May-2020	7-Aug-2020	*Lepus townsendii*	2	+	+	+	v1	–	+
HR20-081	15-Jul-2020	20-Aug-2020	*Lepus townsendii*	2	+	+	+	v1	–	–
HR20-082	21-Jul-2020	20-Aug-2020	*Lepus townsendii*	1	–	–	–	n/a	–	–
HR20-083	20-Jul-2020	20-Aug-2020	*Lepus townsendii*	2	–	–	–	n/a	–	–
HR20-084	14-Jul-2020	21-Aug-2020	*Lepus townsendii*	2	+	–	+	v5	–	–
HR20-085	26-May-2020	21-Aug-2020	*Lepus townsendii*	1	+	+	+	v1	–	–
HR20-086	18-Jun-2020	21-Aug-2020	*Lepus townsendii*	2	–	–	–	n/a	–	–
HR20-087	25-Jun-2020	31-Aug-2020	*Lepus townsendii*	2	–	+	+	v1	–	+
HR20-088	17-Jun-2020	1-Sep-2020	*Lepus townsendii*	1	+	+	+	v1	–	+
HR20-089	8-Jun-2020	1-Sep-2020	*Lepus townsendii*	1	+	+	+	v2	–	+
HR20-090	?-?-2020	1-Sep-2020	*Lepus townsendii*	1	+	+	+	v2	+	+
HR20-091	11-May-2020	3-Sep-2020	*Lepus townsendii*	1	–	+	+	v1	–	–
HR20-092	11-May-2020	3-Sep-2020	*Lepus townsendii*	1	–	–	–	n/a	–	+
HR20-093	7-May-2020	3-Sep-2020	*Lepus townsendii*	2	–	–	–	n/a	–	–
HR20-094	20-Apr-2020	4-Sep-2020	*Lepus townsendii*	1	–	–	–	n/a	–	–
HR20-095	25-Apr-2020	4-Sep-2020	*Lepus townsendii*	2	+	+	+	v1	+	+
HR20-096	11-May-2020	8-Sep-2020	*Lepus townsendii*	2	–	–	–	n/a	–	–
HR20-097	29-Apr-2020	8-Sep-2020	*Lepus townsendii*	1	+	+	+	v1	–	–
HR20-098	30-Apr-2020	8-Sep-2020	*Lepus townsendii*	1	–	–	–	n/a	–	–
HR20-099	27-Apr-2020	11-Sep-2020	*Lepus townsendii*	1	+	+	+	v1	–	+
HR20-100	21-May-2020	14-Sep-2020	*Lepus townsendii*	1	+	+	+	v1	–	+
HR20-101	8-Jun-2020	14-Sep-2020	*Lepus townsendii*	2	+	+	+	v1	–	–
HR20-102	21-Jun-2020	14-Sep-2020	*Lepus townsendii*	2	+	+	+	v1	–	–
HR20-103	10-Jun-2020	15-Sep-2020	*Lepus townsendii*	1	–	+	+	v1	–	–
HR20-104	30-Jun-2020	15-Sep-2020	*Lepus townsendii*	1	+	–	+	v1	–	–
HR20-105	4-Jun-2020	17-Sep-2020	*Lepus townsendii*	1	+	+	+	v1	–	+
HR20-106	1-Jul-2020	17-Sep-2020	*Lepus townsendii*	1	+	+	+	v1	–	+
HR20-107	27-May-2020	17-Sep-2020	*Lepus townsendii*	2	+	+	+	v1	–	+
HR20-108	22-Apr-2020	18-Sep-2020	*Lepus townsendii*	1	+	+	+	v1	+	–
HR20-110	11-Jun-2020	21-Sep-2020	*Lepus townsendii*	1	+	+	+	v1	–	–
HR20-113	11-Jun-2020	21-Sep-2020	*Lepus townsendii*	2	–	–	–	n/a	–	+
HR20-114	8-Jun-2020	21-Sep-2020	*Lepus townsendii*	1	+	+	+	v1	–	+
HR20-115	21-Apr-2020	21-Sep-2020	*Lepus townsendii*	Unknowm	+	+	+	v1	–	–
HR20-117	23-Jun-2020	22-Sep-2020	*Lepus townsendii*	1	+	+	+	v1	–	–
HR20-118	Unknowm	22-Sep-2020	*Lepus townsendii*	1	+	–	+	v1	–	–
HR20-122	29-Apr-2020	24-Sep-2020	*Lepus townsendii*	2	+	+	+	v1	–	–
HR20-123	23-Jun-2020	24-Sep-2020	*Lepus townsendii*	1	+	+	+	v3	–	+
HR20-126	15-May-2020	24-Sep-2020	*Lepus townsendii*	2	+	+	+	v1	+	+
HR20-127	23-Jun-2020	24-Sep-2020	*Lepus townsendii*	1	+	+	+	v2	–	–
HR20-128	9-Jul-2020	25-Sep-2020	*Lepus townsendii*	2	+	+	+	v1	–	–
HR20-129	18-Jun-2020	25-Sep-2020	*Lepus townsendii*	1	+	+	+	v1	–	–
HR20-130	9-Jul-2020	25-Sep-2020	*Lepus townsendii*	2	–	–	–	n/a	–	–
HR20-131	22-Jun-2020	25-Sep-2020	*Lepus townsendii*	2	–	+	+	v1	–	–
HR20-132	3-Jun-2020	25-Sep-2020	*Lepus townsendii*	1	–	–	–	n/a	–	–
HR20-133	9-Jul-2020	5-Oct-2020	*Lepus townsendii*	1	–	–	–	n/a	–	–
HR20-135	6-Jul-2020	5-Oct-2020	*Lepus townsendii*	1	+	+	+	v1	–	–
HR20-137	7-Jul-2020	5-Oct-2020	*Lepus townsendii*	2	–	–	–	n/a	–	–
HR20-140	2-Jul-2020	6-Oct-2020	*Lepus townsendii*	2	+	+	+	v1	–	+
HR20-142	9-Jul-2020	6-Oct-2020	*Lepus townsendii*	1	–	–	–	n/a	–	–
HR20-143	23-Apr-2020	6-Oct-2020	*Lepus townsendii*	2	+	+	+	v1	+	–
HR20-145	23-Apr-2020	8-Oct-2020	*Lepus townsendii*	1	+	+	+	v1	–	+
HR20-146	16-Apr-2020	8-Oct-2020	*Lepus townsendii*	2	+	+	+	v1	–	+
HR20-147	14-Apr-2020	8-Oct-2020	*Lepus townsendii*	1	+	+	+	v1	–	–
HR20-148	20-Apr-2020	8-Oct-2020	*Lepus townsendii*	1	+	+	+	v1	–	+
HR20-149	21-Apr-2020	8-Oct-2020	*Lepus townsendii*	1	+	+	+	v1	–	–
HR20-150	22-Apr-2020	8-Oct-2020	*Lepus townsendii*	1	+	+	+	v2	–	–
HR20-151	28-Apr-2020	9-Oct-2020	*Lepus townsendii*	1	+	+	+	v1	–	+
HR20-154	28-Apr-2020	9-Oct-2020	*Lepus townsendii*	1	+	+	+	v1	+	+
HR20-035	11-Feb-2020	27-Feb-2020	*Oryctolagus cuniculus*	2	–	–	–	n/a	–	–
HR20-042	4-Mar-2020	12-Mar-2020	*Oryctolagus cuniculus*	2	+	+	+	v1	–	–
HR20-054	7-Apr-2020	25-Jun-2020	*Oryctolagus cuniculus*	1	–	–	–	n/a	–	–
HR20-109	11-Jun-2020	21-Sep-2020	*Oryctolagus cuniculus*	1	–	–	–	n/a	–	–
HR20-134	7-Jul-2020	5-Oct-2020	*Oryctolagus cuniculus*	2	+	+	+	v2	–	–
HR20-139	7-Jul-2020	6-Oct-2020	*Oryctolagus cuniculus*	2	–	–	–	n/a	–	–
HR20-152	21-Jul-2020	9-Oct-2020	*Oryctolagus cuniculus*	2	–	–	–	n/a	–	–
HR20-153	24-May-2020	9-Oct-2020	*Oryctolagus cuniculus*	1	–	–	–	n/a	–	–

n/a, not applicable; ?, unidentfied dates or months; mtSSU, mitochondrial small-subunit rRNA gene; mtLSU, mitochondrial large-subunit rRNA gene; dhps, dihydropteroate synthase; ITS1-5.8s-ITS2, nuclear internal transcribed spacer 1, 5.8S rRNA and internal transcribed spacer 2.
