# Supplementary material for: Development of Highly Efficient Universal Pneumocystis Primers and Their Application in Investigating the Prevalence and Genetic Diversity of Pneumocystis in Wild Hares and Rabbits
Source: J Fungi (Basel). 2024 May 15;10(5):355. doi: 10.3390/jof10050355 (PMC11121927; doi:10.3390/jof10050355)
Supplement: Supplementary file 1 [file jof-10-00355-s001.zip › Table S2_PCR primers.pdf]

**Supplemental Table S2. PCR primers used in this study.**

| Names | Sequences (5′→ 3′)[a] | Targets | References |
|---|---|---|---|
| SSU.f1 | CCTACAGCTACCTTATTTCGA | mtSSU | [35] |
| SSU.r1 | ATGRAGTGGGCTACAGACGTG | mtSSU | [35] |
| rns.f4 | TTCAGCTACAGGTTCC | mtSSU | This study |
| rns.f6b | YRGCACGTCTGTAGCCCACT | mtSSU | This study |
| rns.f9 | TTCCCCTACAGCTACCTTA | mtSSU | This study |
| rns.f8 | GCGCTTGACGAGTAGTTAGT | mtSSU | This study |
| rn3.r3 | ATCTGTGATGTACTAACTAC | mtSSU | This study |
| rns.r10 | CAAAACATTAGACGGTCACAG | mtSSU | This study |
| rns.r13 | AGATACCCTTGTAGTTTATGCTG | mtSSU | This study |
| rnl.r8 | AGGAAAAGAAATCAACCGAGA | mtLSU | This study |
| LSU.f1 | CTCATGTCAGCATTCTCTCTTTA | mtLSU | This study |
| LSU.r1 | TAGGATATAGCTGGTTTTCTGCGA | mtLSU | This study |
| rnl.f1c | CCCAACTCACGTAACCTTTTA | mtLSU | This study |
| rnl.f3 | TTTGCCGAGTTCCTT | mtLSU | This study |
| rnl.f10 | AACGTTACTCATGTCAGCA | mtLSU | This study |
| rnl.f11 | TACTAAATGTTCTGCTACC | mtLSU | This study |
| rnl.r4 | GGTTACGTGAGTTGGGTT | mtLSU | This study |
| rnl.r12 | ACCGTGAGGGAAAGTTGA | mtLSU | This study |
| Pu3Y.f[b] | TACAAATCGRACTAGGATAT | mtLSU | This study |
| Pu7rev | GATGGCTGTTTCCAAG | mtLSU | [33] |
| pAZ102-E | GATGGCTGTTTCCAAGCCCA | mtLSU | [34] |
| pAZ102-H | GTGTACGTTGCAAAGTACTC | mtLSU | [34] |
| nad5.r6 | CCWAWWGMWACAAACATCATWCC | *nad5* | This study |
| nad5.f6 | TGTTCWCARTTRGGWATGATGTTTGT | *nad5* | This study |
| cob.r8 | GGATTAGCCATRATRTAATTATCRCTATG | *cob* | This study |
| cob.f8 | CATAGYGATAATTAYATYATGGCTAATCC | *cob* | This study |
| rns.cox1.f5 | GGWACTARCCAATTWCCAAATC | *cox1* | This study |
| cox1.r5 | TDGGWGGATTTGGWAATTGG | *cox1* | This study |
| mt.r103c | GCATCCWTCAAYGTGACAAATTGA | mtDNA non-coding region | This study |
| dhps.f7 | GGTGTTCATTCATATGATTC | *dhps* | This study |
| dhps.f6 | CCRRTTTTRCCATTYTTA | *dhps* | This study |
| dhps.r6 | CCTCTCATRTGCATTATACATATYGG | *dhps* | This study |
| dhps.r7 | AYCCTGTAAAYCTTTT | *dhps* | This study |
| dhps.r8 | CCAGGRTCYAAAATAATRTTCCA | *dhps* | This study |
| dhps.r9 | AATAWAHYYYTTTCCARATAGCATC | *dhps* | This study |
| PK.f1 | CAARTTRTCAGAAGAAAAGATGGT | *pppk* | This study |
| PK.f2 | TRAAAACRTCTATGTTRTATGAATC | *pppk* | This study |
| PK160 | GTTAATCCTGGTATTAAACCAGTTTTGCCATT | *pppk* | [32] |
| 16S.f4 | CGCTACTACCGATTGAATGGC | 18S rRNA | This study |
| ITS1.F | TTRCTGGRAAGTTGATCAAAT | 18S rRNA | This study |

| | | | |
|---|---|---|---|
| ITS1-1f | TCAAATTTGGTCATTTAGAGG | 18S rRNA | This study |
| 26S.r1 | TGCCACGTTCTTCATCGAC | 5.8S rRNA | This study |
| Fun.ITS4 | TCCTCCGCTTATTGATATGC | 28S rRNA | [36] |
| 26S.r8 | GGCTTAATCTCAGCAGATC | 28S rRNA | This study |
| 16SrRNA.r1 | CTGGCTTCACCCTATTCAGG | 28S rRNA | This study |
| PCP18S.f3 | GAATGGCTCATTATATCAGTTATA | 18S rRNA | This study |
| Nu26S.f9 | CACGGACCAAGGAGTCTAATA | 28S rRNA | This study |
| PCP28S.r1 | CAARGGCTTAATCTCAGCAGA | 28S rRNA | This study |
| CTB.f3 | ATGACCAACATYCGYAAAAC | *cytb* | This study |
| CTB.r3 | AATGATATTTGBCCTCATGG | *cytb* | This study |

[a] Non-standard nucleotides represent degenerate codes. All primers are for *Pneumocystis* except for the last two (CTB.f3 and CTB.r3) which are shared between the host hares and rabbits.

[b] Modified from Riedbold *et al.* [32] by incorporating a degenerate nucleotide R at the 10th position.

Abbreviations: mtSSU, mitochondrial small-subunit rRNA gene; mtLSU, mitochondrial large-subunit rRNA gene; *cob*, apocytochrome b; *nad5*, NADH dehydrogenase subunit 5; *cox1*, cytochrome oxidase subunit 1; *cytb*, cytochrome b gene (from mammals), mtDNA, mitochondrial genome DNA; *dhps*, dihydropteroate synthase gene; *pppk*, dihydro-6-hydroxymethylpterin pyrophosphokinase gene; ITS1-5.8S-ITS2, nuclear internal transcribed spacer 1, 5.8S rRNA and internal transcribed spacer 2.
