# Supplementary material for: Development of Highly Efficient Universal Pneumocystis Primers and Their Application in Investigating the Prevalence and Genetic Diversity of Pneumocystis in Wild Hares and Rabbits
Source: J Fungi (Basel). 2024 May 15;10(5):355. doi: 10.3390/jof10050355 (PMC11121927; doi:10.3390/jof10050355)
Supplement: Supplementary file 1 [file jof-10-00355-s001.zip › Table S3_Samples for primer tests.pdf]

Supplementary Table S3 ***Pneumocystis* samples used for testing universal primers.**

Host common names	Host scientific names	*Pneumocystis* species	Sampling countries & years	Sample type	References
Human	*Homo sapiens*	*P. jirovecii*	USA, 1986	Frozen lung	[32]
House mouse	*Mus musculus*	*P. murina*	USA, 2012	Frozen lung	[30]
Prairie dog	*Cynomys ludovicianus*	*P. ludoviciani*	USA, 2010	FFPE	[42]
Rhesus macaque	*Macaca mulatta*	*P. macacae*	USA, 2018	Frozen lung	[24]
Ferret	*Mustela putorius furo*	*Pneumocystis* spp.	USA, 1999	Frozen lung	[43]
Hare	*Lepus townsendii*	*P.* sp. '*townsendii*'	Canada, 2020	Frozen lung	This study
Brown rat	*Rattus norvegicus*	*P. carinii*	Austria, 2004	FFPE lung	[35]
European rabbit	*Oryctolagus cuniculus*	*P. oryctolagi*	Austria, 2008	FFPE lung	[35]
Whippet dog	*Canis lupus familiaris*	*P. canis*	Austria, 2013	FFPE lung	[44]
Pig	*Sus scrofa domesticus*	*Pneumocystis* spp.	Austria, 2019	Frozen lung	[35]
Goat	*Capra aegagrus hircus*	*Pneumocystis* spp.	Austria, 2017	FFPE lung	[35]
Shrew	*Neomys anomalus*	*Pneumocystis* spp.	Austria, 2015	FFPE lung	[37]
River rat	*Myocastor coypus*	*Pneumocystis* spp.	Italy, 2013-14	Frozen lung	[41]
Squirrel	*Callosciurus finlaysonii*	*Pneumocystis* spp.	Italy, 2014	Frozen lung	[41]
Cat	*Felis catus*	*Pneumocystis* spp.	Italy, 2017-18	Frozen lung	[40]

FFPE, formalin-fixed, paraffin-embedded; mtLSU, mitochondrial large-subunit rRNA gene; mtSSU, mitochondrial small-subunit rRNA gene. *Indicating sequences identical to existing sequences in GenBank, with one accession no. shown if both genes are included in the mitochondrial genome sequence.
