# Supplementary material for: Development of Highly Efficient Universal Pneumocystis Primers and Their Application in Investigating the Prevalence and Genetic Diversity of Pneumocystis in Wild Hares and Rabbits
Source: J Fungi (Basel). 2024 May 15;10(5):355. doi: 10.3390/jof10050355 (PMC11121927; doi:10.3390/jof10050355)
Supplement: Supplementary file 1 [file jof-10-00355-s001.zip › Table S4_Sequence identity matrix.pdf]

Supplemental Tables S4. Nucleotide identity and similarity among *Pneumocystis* from different animals for the dihydropteroate synthase (*dhps*) gene, nuclear rRNA operon (rDNA) and mitochondrial genome DNA (mtDNA).

***dhps* sequence identity and similarity (~1.3 kb aligned length*)**

Identity Scores (%), above the diagonal

	Ph_v1	Ph_v2	Pory	Pcan	Pmac	Pjir	Pcar	Pwak	Pmur
Ph_v1	—	98.4	92.2	82.2	80.8	82.7	80.2	79.4	80.6
Ph_v2	98.4	—	92.2	81.9	80.9	82.1	80.3	79.3	80.8
Pory	92.2	92.2	—	83.7	83.3	83.7	80.9	80.4	81.3
Pcan	82.2	81.9	83.7	—	83.1	84.6	82.9	82.3	82.8
Pmac	80.8	80.9	83.3	83.1	—	88.0	82.0	81.1	81.9
Pjir	82.7	82.1	83.7	84.6	88.0	—	81.9	81.4	82.9
Pcar	80.2	80.3	80.9	82.9	82.0	81.9	—	93.7	93.9
Pwak	79.4	79.3	80.4	82.3	81.1	81.4	93.7	—	94.9
Pmur	80.6	80.8	81.3	82.8	81.9	82.9	93.9	94.9	—

Similarity Scores (%), below the diagonal

*Including a small portion of the dihydro-6-hydroxymethylpterin pyrophosphokinase (pppk) gene upstream of the *dhps* gene (Figure S5).

rDNA sequence identity and similarity (~6.4 kb aligned length)

Identity Scores (%) , above the diagonal

	Ph_v1	Ph_v2	Pory	Pcan	Pmac	Pjir	Pcar	Pwak	Pmur
Ph_v1	—	99.2	96.3	80.3	79.3	86.2	78.8	84.3	79.1
Ph_v2	99.2	—	96.5	80.5	79.3	86.2	78.9	84.3	79.1
Pory	96.3	96.5	—	80.3	79.1	86.2	78.7	84.0	79.1
Pcan	80.3	80.5	80.3	—	85.5	83.8	85.4	82.3	86.3
Pmac	79.3	79.3	79.1	85.5	—	85.6	84.3	81.0	85.0
Pjir	86.2	86.2	86.2	83.8	85.6	—	82.1	87.7	82.9
Pcar	78.8	78.9	78.7	85.4	84.3	82.1	—	88.0	94.0
Pwak	84.3	84.3	84.0	82.3	81.0	87.7	88.0	—	89.0
Pmur	79.1	79.1	79.1	86.3	85.0	82.9	94.0	89.0	—

Similarity Scores (%), below the diagonal

mtDNA sequence identity and similarity (~19.3 kb aligned length*)

Identity Scores (%) , above the diagonal

	Ph_v1	Ph_v2	Pory	Pcan	Pmac	Pjir	Pcar	Pwak	Pmur
Ph_v1	—	94.7	84.7	71.6	70.2	69.0	66.6	67.3	69.5
Ph_v2	94.7	—	84.3	71.4	70.2	68.8	66.7	67.2	69.4
Pory	84.7	84.3	—	72.9	71.2	69.3	67.3	68.3	70.4
Pcan	71.6	71.4	72.9	—	73.2	70.3	69.3	69.7	71.8
Pmac	70.2	70.2	71.2	73.2	—	76.5	67.7	68.6	70.1
Pjir	69.0	68.8	69.3	70.3	76.5	—	65.9	66.1	68.4
Pcar	66.6	66.7	67.3	69.3	67.7	65.9	—	75.7	78.0
Pwak	67.3	67.2	68.3	69.7	68.6	66.1	75.7	—	80.6
Pmur	69.5	69.4	70.4	71.8	70.1	68.4	78.0	80.6	—

Similarity Scores (%) , below the diagonal

*Including 15 protein-coding genes and 3 rRNA genes concatenated for sequence alignment. The names of *Pneumocystis* species and variants are abbriavated as follows: Ph_v1, *P.* sp. *'townsendii'* variant 1; Ph_v2, *P.* sp. *'townsendii'* variant 2; *Pory, P. oryctolagi; Pcan, P. canis; Pmac, P. macacae; Pjir, P. jirovecii; Pcar, P. carinii; Pwak, P. wakefieldiae; Pmur, P. murina.* GenBank accession numbers for these sequences are listed in Table S6.
