# Supplementary material for: Development of Highly Efficient Universal Pneumocystis Primers and Their Application in Investigating the Prevalence and Genetic Diversity of Pneumocystis in Wild Hares and Rabbits
Source: J Fungi (Basel). 2024 May 15;10(5):355. doi: 10.3390/jof10050355 (PMC11121927; doi:10.3390/jof10050355)
Supplement: Supplementary file 1 [file jof-10-00355-s001.zip › Table S5_Sequence distance matrix.pdf]

Supplemental Tables S5. Pairwise genetic distance among *Pneumocystis* from different animals at the dihydropteroate synthase (*dhps*) gene, nuclear rRNA operon (rDNA) and mitochondrial genome DNA (mtDNA).

Genetic distance based on *dhps* nucleotide sequence (~1.3 kb aligned length*)

	Ph_v1	Ph_v2	Pory	Pcan	Pmac	Pjir	Pcar	Pwak
Ph_v2	0.0162							
Pory	0.0680	0.0680						
Pcan	0.1808	0.1847	0.1779					
Pmac	0.2051	0.2031	0.1901	0.1877				
Pjir	0.1733	0.1801	0.1782	0.1717	0.1281			
Pcar	0.2102	0.2091	0.2173	0.1906	0.2023	0.2010		
Pwak	0.2222	0.2233	0.2253	0.1986	0.2163	0.2091	0.0665	
Pmur	0.2059	0.2029	0.2120	0.1906	0.2051	0.1880	0.0632	0.0524

*Including a small portion of the dihydro-6-hydroxymethylpterin pyrophosphokinase (pppk) gene upstream of the *dhps* gene (Figure S5).

Genetic distance based on rDNA sequence (~6.4 kb aligned length)

	Ph_v1	Ph_v2	Pory	Pcan	Pmac	Pjir	Pcar	Pwak
Ph_v2	0.0079							
Pory	0.0273	0.0248						
Pcan	0.0756	0.0716	0.0760					
Pmac	0.0738	0.0726	0.0740	0.1089				
Pjir	0.0690	0.0680	0.0698	0.0900	0.0637			
Pcar	0.0823	0.0813	0.0841	0.1174	0.1223	0.1034		
Pwak	0.0866	0.0859	0.0884	0.1024	0.1042	0.1022	0.0482	
Pmur	0.0843	0.0824	0.0844	0.1073	0.1158	0.0950	0.0541	0.0501

Genetic distance based on mtDNA (~19.3 kb aligned length*)

	Ph_v1	Ph_v2	Pory	Pcan	Pmac	Pjir	Pcar	Pwak
Ph_v2	0.0520							
Pory	0.1500	0.1546						
Pcan	0.3048	0.3095	0.2947					
Pmac	0.3118	0.3132	0.3036	0.2775				
Pjir	0.3275	0.3307	0.3251	0.3122	0.2212			
Pcar	0.3424	0.3437	0.3341	0.3107	0.3226	0.3466		
Pwak	0.3357	0.3397	0.3279	0.3074	0.3176	0.3517	0.2097	
Pmur	0.3199	0.3236	0.3079	0.2966	0.3055	0.3434	0.1933	0.1604

*Including 15 protein-coding genes and 3 rRNA genes concatenated for sequence alignment.
The names of *Pneumocystis* species and variants are abbriavated as follows: Ph_v1, *P.* sp. *'townsendii'* variant 1; Ph_v2, *P.* sp. *'townsendii'* variant 2; *Pory, P. oryctolagi; Pcan, P. canis; Pmac, P. macacae; Pjir, P. jirovecii; Pcar, P. carinii; Pwak, P. wakefieldiae; Pmur, P. murina.* GenBank accession numbers for these sequences are listed in Table S6.
