# Supplementary material for: Development of Highly Efficient Universal Pneumocystis Primers and Their Application in Investigating the Prevalence and Genetic Diversity of Pneumocystis in Wild Hares and Rabbits
Source: J Fungi (Basel). 2024 May 15;10(5):355. doi: 10.3390/jof10050355 (PMC11121927; doi:10.3390/jof10050355)
Supplement: Supplementary file 1 [file jof-10-00355-s001.zip › Table S6_GenBank Nos.pdf]

Supplemental Table S6. GenBank accession numbers for *Pneumocystis* sequences involved in this study.

Species and strains	mtLSU	mtSSU	mtDNA	rDNA	*dhps*
P. sp. *'townsendii'* variant 1	PP477327	PP477339	PP484880	PP476603	PP477274
P. sp. *'townsendii'* variant 2	PP477328	PP477340	PP484881	PP476604	PP477275
P. sp. *'townsendii'* variant 3	PP477329	PP477341	n/a	n/a	n/a
P. sp. *'townsendii'* variant 4	n/a	PP477342	n/a	n/a	n/a
P. sp. *'townsendii'* variant 5	n/a	PP477343	n/a	n/a	n/a
P. oryctolagi variant 1	PP477330	PP477344	n/a	n/a	n/a
P. oryctolagi variant 2	PP477331	PP477344	n/a	n/a	n/a
P. oryctolagi RABM	MT726213*	MT726213*	MT726213†	MT780543†	PORY_002119†
P. canis AU	MT726217*	MT726217*	MT726217†	MT780538†	PCANB_001404†
P. macacae P2C	MT726214*	MT726214*	MT726214†	MT780541†	PMAC_002379†
P. jirovecii RU7	NC_020331*	NC_020331*	NC_020331†	MT780540†	T551_02230†
P. carinii B80	JX499145*	JX499145*	JX499145†	MT780539†	T552_03074†
P. wakefieldiae 2A	MT726211	MT726211*	MT726211†	MT780544†	MERGE_001568†
P. murina B123	JX499144*	JX499144*	JX499144†	MT780542†	PNEG_03560†
*P. ludoviciani*ϕ	MT726212*	MT726212*	MT726212	PP476606	PP477272
Pneumocystis in squirrel	PP484879*	PP484879*	PP484879	PP476605	PP477273
Pneumocystis in ferret	P477333	PP477345	n/a	n/a	n/a
Pneumocystis in pig	P477334	PP477346	n/a	n/a	n/a
Pneumocystis in goat	P477337	PP477349	n/a	n/a	n/a
Pneumocystis in shrew	P477336	PP477348	n/a	n/a	n/a
Pneumocystis in river rat	P477335	PP477347	n/a	n/a	n/a
Pneumocystis in cat	P477338	PP477350	n/a	n/a	n/a

* Included in the respective mtDNA sequences.

† From previous genome studies by Ma *et al.* [30] and Cisse *et al*. [24], with the *dhps* sequences available from GenBank using the locus tags listed.

ϕ *Pneumocystis* from the black-tailed prairie dog recently registered in MycoBank with ID: MBT10019263 (https://www.mycobank.org).

Abbreviations: mtSSU, mitochondrial small-subunit rRNA gene; mtLSU, mitochondrial large-subunit rRNA gene; mtDNA, mitochondrial genome DNA; rDNA, nuclear rRNA operon; *dhps*, dihydropteroate synthase gene; n/a, not available.
